# Prolactin and its receptor as therapeutic targets in glioblastoma multiforme

**DOI:** 10.1038/s41598-019-55860-x

**Published:** 2019-12-20

**Authors:** Antonela Sofía Asad, Alejandro Javier Nicola Candia, Nazareno Gonzalez, Camila Florencia Zuccato, Araceli Abt, Santiago Jordi Orrillo, Yael Lastra, Emilio De Simone, Florence Boutillon, Vincent Goffin, Adriana Seilicovich, Daniel Alberto Pisera, María Jimena Ferraris, Marianela Candolfi

**Affiliations:** 1 Instituto de Investigaciones Biomédicas (INBIOMED, UBA-CONICET), Facultad de Medicina, Universidad de Buenos Aires, Buenos Aires, Argentina; 2 Departamento de Biología Celular e Histología, Facultad de Medicina, Universidad de Buenos Aires, Buenos Aires, Argentina; 3 Max Planck Laboratory for Structural Biology, Chemistry and Molecular Biophysics of Rosario (MPLbioR), Universidad Nacional de Rosario, Rosario, Argentina; 4 Cátedra de Fisiología Animal, Facultad de Ciencias Veterinarias, Universidad de Buenos Aires, Buenos Aires, Argentina; 5 Inserm U1151, Institut Necker Enfants Malades (INEM), Faculty of Medicine, University Paris Descartes, Paris, France

**Keywords:** Cancer models, CNS cancer

## Abstract

Although prolactin (PRL) and its receptor (PRLR) have been detected in glioblastoma multiforme (GBM), their role in its pathogenesis remains unclear. Our aim was to explore their contribution in GBM pathogenesis. We detected PRL and PRLR in all GBM cell lines tested. PRLR activation or overexpression using plasmid transfection increased proliferation, viability, clonogenicity, chemoresistance and matrix metalloproteinase activity in GBM cells, while PRLR antagonist ∆1–9-G129R-hPRL reduced their proliferation, viability, chemoresistance and migration. Meta-analysis of transcriptomic data indicated that PRLR was expressed in all grade II-III glioma (GII-III) and GBM samples. PRL was upregulated in GBM biopsies when compared to GII-III. While in the general population tumour PRL/PRLR expression did not correlate with patient survival, biological sex-stratified analyses revealed that male patients with PRL^+^/PRLR^HIGH^ GBM performed worse than PRL^+^/PRLR^LOW^ GBM. In contrast, all male PRL^+^/PRLR^HIGH^ GII-III patients were alive whereas only 30% of PRL^+^/PRLR^LOW^ GII-III patients survived after 100 months. Our study suggests that PRLR may be involved in GBM pathogenesis and could constitute a therapeutic target for its treatment. Our findings also support the notion that sexual dimorphism should be taken into account to improve the care of GBM patients.

## Introduction

Gliomas are primary tumours of the central nervous system (CNS) that develop from glial cells. While grade I gliomas are non-infiltrative tumours that are usually cured with complete surgical resection, grade II-IV gliomas are highly invasive, which eventually leads to the death of the patients^1^. More than half of these gliomas are grade IV, otherwise known as glioblastomas multiforme (GBM), for which the overall 5-year survival rate remains below 5%^1^. Many therapeutic challenges characterize GBM, i.e. the invasion within the non-neoplastic brain, which makes complete tumour resection virtually impossible; the intrinsic resistance of GBM cells to chemotherapy and radiotherapy; and the immunosuppressive microenvironment, which impairs the development of an adequate antitumour immune response. Thus, it is crucial to better understand the pathogenesis of this disease and to identify novel therapeutic targets that could improve the treatment of GBM patients.

Prolactin (PRL) is a peptide hormone primarily secreted by the anterior pituitary gland. Although PRL has been traditionally associated principally to the regulation of lactation and fertility, this hormone has been lately involved in the development of several types of cancer^2,3^. Extrapituitary sources of PRL include the mammary gland, prostate, brain, immune cells and skin, where this hormone acts as a paracrine/autocrine physiological regulator of tissue development and homeostasis. In addition, PRL and its receptor (PRLR) have been associated with the development of hormone-dependent tumours, such as breast and prostate cancer^2,3^. The expression of PRL and PRLR has been reported to be higher in breast and prostate cancer than in their healthy counterparts and has been associated with increased risk of breast and prostate cancer and treatment resistance^2^. PRL has also been reported to promote tumour cell proliferation, angiogenesis^4–6^ and chemoresistance^7,8^. Although there is controversy on the role of PRL/PRLR in the pathogenesis of breast tumours^3^, blockade of PRLR has been proposed to constitute a therapeutic approach for the treatment of hormone-dependent tumours, including breast cancer. Since extrapituitary expression of PRL is independent of dopamine regulation, the use of PRLR antagonists or neutralizing antibodies and small molecule inhibitors has been proposed as therapeutic alternatives for breast and prostate resistant tumours^2,3^.

In humans, various isoforms of the PRLR, i.e. long, intermediate and short, result from alternative splicing and vary in the length of the intracellular domain^9^. Since they encompass different signalling pathways, the relative expression of these receptors in normal and pathological tissues may explain, in part, the versatility of PRL actions reported in several tissues, such as the healthy and neoplastic mammary gland^3^. Although many reports have indicated that PRL and PRLR are present in GBM^10–13^, the role of the PRL/PRLR system in the pathogenesis of GBM remains poorly understood. The expression of PRL and PRLR has been detected in GBM biopsies by immunocytochemistry^10–13^. Furthermore, high levels of circulating PRL have been reported in ~30% of GBM patients in a relatively small clinical study^11^. The expression levels of the PRL gene have also been reported to be higher in GBM than in GII-III^14^. In addition, local expression of PRL and high levels of circulating PRL have been shown to correlate with the proliferation index and the vascular density of GBM^11^. PRL was reported to modulate the expression of intercellular adhesion molecules^15^ and to facilitate the migration of human GBM cells *in vitro*^12^. Activation as well as overexpression of PRLR have been shown to stimulate the proliferation of GBM cell lines *in vitro*^16^.

In order to shed light on the role of PRL and PRLR in the pathogenesis of GBM, we evaluated proliferation, viability, chemosensitivity and migration of GBM cells in response to PRL stimulation or PRLR signalling blockade using the receptor-specific antagonist ∆1–9-G129R-hPRL (PRLR-A)^17^, and to the overexpression of the long and short isoforms of PRLR. We also performed bioinformatics analysis of PRL and PRLR transcriptomic data from GII-III and GBM patients and its correlation with survival. Our findings suggest that the activation of the PRL/PRLR pathway may facilitate GBM tumour progression.

## Results

### PRL and PRLR are expressed in GBM cells

**Figure 1:**
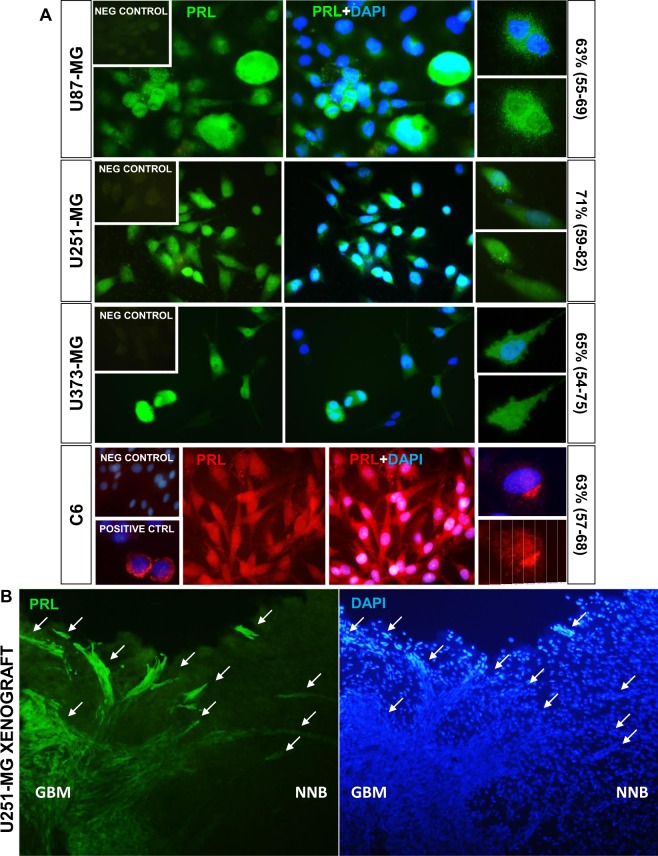
Expression of prolactin in experimental glioblastoma multiforme. (**A**) Representative low and high magnification microphotographs show PRL expression in human (U87-MG, U251-MG and U373-MG, green staining) and rat (C6, red staining) GBM cell lines, as assessed by immunofluorescence using specific antibodies against human and rat PRL. Insets show representative negative controls and a positive control (somatolactotrope GH3 cells). Nuclei were stained with DAPI. The percentage of PRL^+^ cells (and confidence intervals) for each of the cell lines are depicted on the right side of each panel. (**B**) Human PRL expression (green fluorescence) was assessed in brain sections from nude mice bearing intracranial U251-MG human GBM xenografts. A representative field is shown. NNB: non-neoplastic brain. Arrows indicate PRL^+^ tumour cells.

We evaluated the expression of PRL in human and rodent GBM cells by immunofluorescence. Human (U251-MG, U87-MG, U373-MG) and rat (C6) GBM cells presented PRL staining (Fig. 1A). We also assessed the content of PRL in cell protein extracts and supernatants from rat and human GBM cells by radioimmunoassay (RIA). While PRL was detected in the supernatant of C6 GBM cells (6–22 ng/ml), PRL levels in the supernatant of U251-MG cells were under the detection threshold. In C6 cell extracts PRL content was between 23.4–38.2 ng/ml, whereas in U251 it was between 0.1 and 0.6 ng/ml. These expression levels are not uncommon for extrapituitary tissues producing PRL, in which local PRL levels are usually below detection limits^18^. The expression of PRL was also evaluated in U251-MG human GBM xenografts growing in the brain of nude mice. PRL^+^ cells were readily detected in tumour cells within the tumour mass as well as in those infiltrating the non-neoplastic brain parenchyma (Fig. 1B).

**Figure 2:**
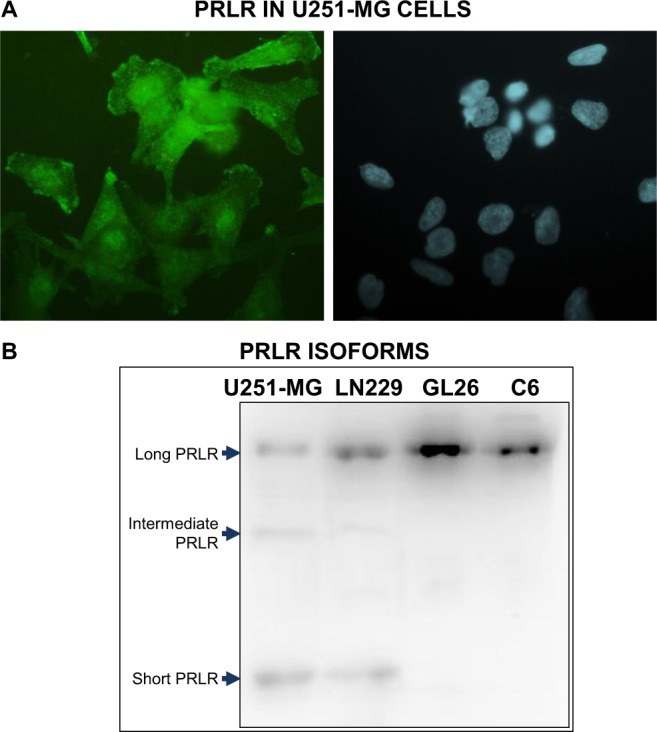
Expression of prolactin receptor in glioblastoma multiforme cells. (**A**) A representative microphotograph shows PRLR expression in human U251-MG GBM cells, as assessed by immunofluorescence using a specific anti-human PRLR (green fluorescence). (**B**) A representative blot shows PRLR isoforms, as evaluated by WB in protein extracts from human (U251-MG, LN229), mouse (GL26) and rat (C6) GBM cells.

PRLR expression was detected in human U251-MG cells by immunofluorescence (Fig. 2A). Since there are several isoforms of PRLR with distinct downstream mechanisms, we assessed their expression in GBM cells by WB (Fig. 2B, Supp. Fig. S1). We detected expression of PRLR in human (U251-MG, LN229), mouse (GL26) and rat (C6) GBM cells. While the long PRLR isoform was detected in cells from all species studied, other bands that presumably corresponded to intermediate and short PRLR isoforms were only observed in human GBM cells.

### PRLR pathway activation enhances proliferation, chemoresistance and migration of GBM cells

**Figure 3:**
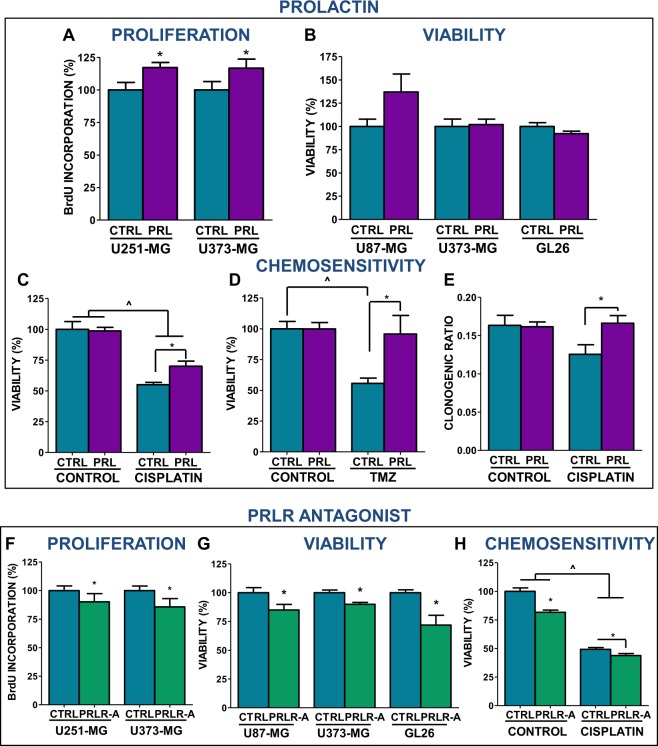
Effect of prolactin or its receptor blockade on proliferation, viability and chemoresistance of glioblastoma multiforme cells. (**A**) Human U251-MG and U373-MG GBM cells were incubated for 6 h with PRL (100 ng/ml) and proliferation was evaluated by BrdU incorporation ELISA. *p < 0.05 vs. respective control (Student’s *t* test). (**B**) The viability of U87-MG, U373-MG and GL26 cells was assessed 72 h after incubation with PRL by MTT assay. (**C**) U251-MG cells were incubated with PRL and cisplatin (5 µM) for 72 h. Cell viability was evaluated by MTT assay. *p < 0.05 vs. respective control without PRL, ^p < 0.05 vs. respective control without cisplatin (ANOVA). (**D**) U251-MG cells were incubated with PRL and TMZ (15 µM) for 72 h. Cell viability was evaluated by MTT assay. *p < 0.05 vs. respective control without PRL, ^p < 0.05 vs. respective control without TMZ (ANOVA). (**E**) Rat GBM cells (C6) were incubated with PRL and 16 h later they were treated with cisplatin (1 µM) for additional 24 h. Cells were then processed for the clonogenic assay. *p < 0.05 vs. respective control without PRL (ANOVA). (**F**) U251-MG and U373-MG GBM cells were incubated for 6 h with PRLR-A (∆1–9-G129R-hPRL, 2.5 µg/ml) and proliferation was evaluated by BrdU incorporation ELISA. *p < 0.05 vs. respective control (Student’s *t* test). (**G**) The viability of U87-MG, U373-MG and GL26 cells was assessed 72 h after incubation with PRLR-A by MTT assay. *p < 0.05 vs. respective control (Student’s *t* test). H) U251-MG cells were incubated with PRLR-A and cisplatin (5 µM) for 72 h. Cell viability was evalua*t*ed by MTT assay. *p < 0.05 vs. respective control without PRLR-A, ^p < 0.05 vs. respective control without cisplatin (ANOVA).

We assessed the effect of PRLR pathway activation or blockade on proliferation, viability and chemosensivity of GBM cells. Addition of PRL (100 ng/ml) stimulated the proliferation of human U251-MG and U373-MG GBM cells (Fig. 3A). However, cell viability was not affected by the incubation with PRL in U87-MG and U373-MG, nor in GL26 mouse GBM cells (Fig. 3B). The cytotoxic effect of chemotherapeutic drug cisplatin on the viability of U251-MG human GBM cells was partially impaired by concomitant treatment with PRL (Fig. 3C). A similar effect was observed when cell death was induced by temozolomide (TMZ, Fig. 3D). Treatment with cisplatin also inhibited the clonogenic response of rat C6 GBM cells, an effect that was inhibited by the presence of PRL (Fig. 3E). On the other hand, PRLR blockade using antagonist ∆1–9-G129R-hPRL (PRLR-A, 2.5 µg/ml) significantly reduced the proliferation rate of U373-MG and U251-MG cells (Fig. 3F), as well as the viability of U87-MG, U373-MG and GL26 cells (Fig. 3G). In addition, PRLR-A increased the cytotoxic effect of cisplatin in U251-MG cells (Fig. 3H).

**Figure 4:**
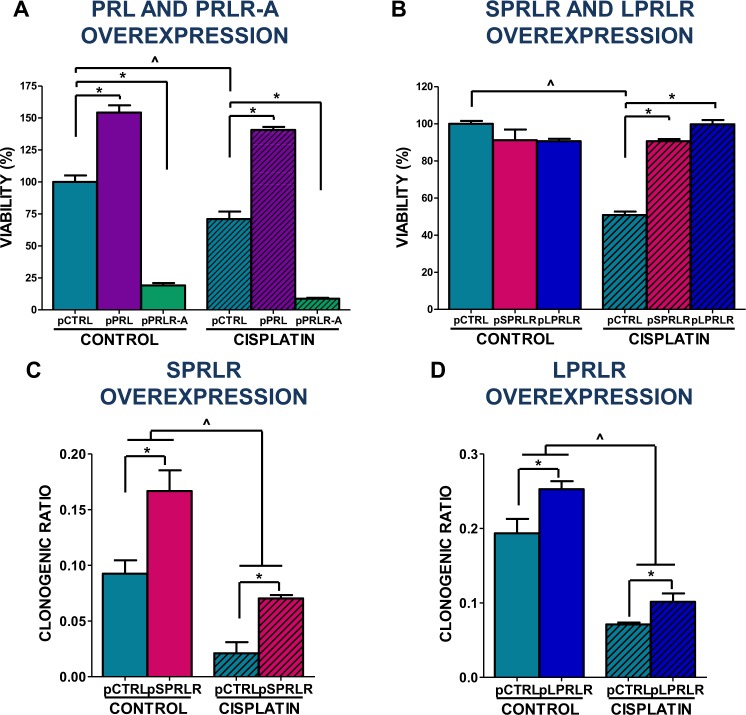
Effect of the overexpression of prolactin, its receptor or the receptor antagonist on the response of glioblastoma multiforme cells to chemotherapy. (**A**) Human U251-MG GBM cells were transfected for 6 h with plasmids encoding human PRL (pPRL) or PRLR-A (pPRLR-A). 16 h later, they were incubated with cisplatin (5 µM) for 72 h. Cell viability was then evaluated by MTT assay. (**B**) Mouse GL26 GBM cells were transfected for 6 h with plasmids encoding the short (pSPRLR) or the long (pLPRLR) isoforms of the mouse PRLR. 16 h later, they were treated with cisplatin (2 µM) for 72 h. Cell viability was assessed by MTT. *p < 0.05 vs. respective control plasmid (pCTRL), ^p < 0.05 vs. respective control without cisplatin (ANOVA). C-D) Rat GBM cells (C6) were transfected for 6 h with plasmids encoding the (**C**) short (pSPRLR) or the (**D**) long (pLPRLR) isoforms of the rat PRLR. 16 h later, they were incubated with cisplatin for additional 24 h. Cells were then processed for the clonogenic assay. *p < 0.05 vs. respective pCTRL, ^p < 0.05 vs. respective control without cisplatin (ANOVA).

We next evaluated the effect of PRL, PRLR or PRLR-A overexpression on the viability and chemosensitivity of GBM cells. While the transfection of U251-MG GBM cells with a plasmid encoding human PRL resulted in increased viability and reduced cytotoxic effect of cisplatin, transfection with a plasmid encoding PRLR-A reduced GBM cell viability and boosted the sensitivity to cisplatin (Fig. 4A). On the other hand, transfection of GL26 GBM cells with plasmids encoding the long or short isoforms of murine PRLR did not directly affect their viability, but both inhibited the cytotoxic effect of cisplatin, as assessed by MTT assay (Fig. 4B). Furthermore, the cytotoxic effect of cisplatin on the clonogenic response of C6 cells was partially impaired by the overexpression of the short (Fig. 4C) and long (Fig. 4D) isoforms of rat PRLR.

**Figure 5:**
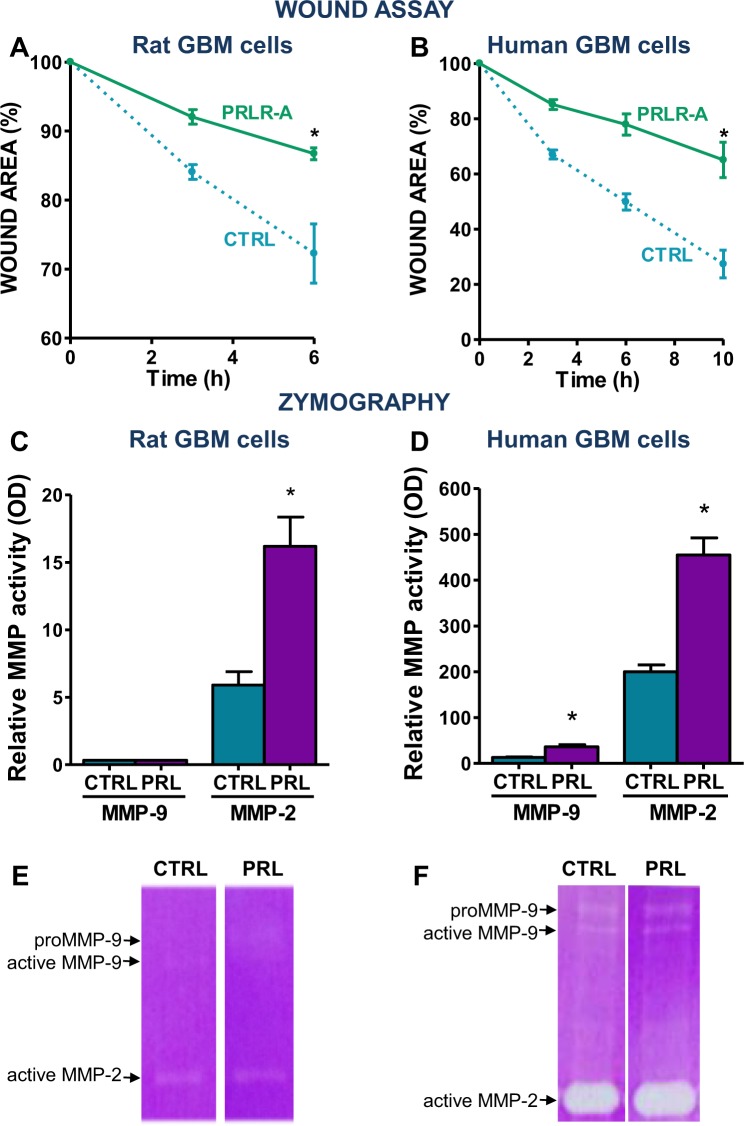
Effect of prolactin receptor blockade on glioblastoma multiforme cell migration. (**A**) Rat (C6) and (**B**) human (LN229) GBM cells were cultured until confluence with PRLR-A (2.5 µg/ml). A scratch was performed in the monolayer and the scratch area was measured at different time points. *p < 0.05 vs. control (Non-lineal regression analysis). Each dot indicates the mean ± SEM of 2 wells. The graphs shown are representative of 3 experiments. (**C,D**) SDS-PAGE gelatine zymography of conditioned media from (**C**) rat and (**D**) human GBM cells incubated in the presence of PRL (100 ng/ml) for 48 h. Gels were stained with Coomassie blue and bands were analysed by densitometry with ImageJ software. Zymographic activity was expressed as percentage in relation to a standard internal sample that saturates at a density of 50%. *p < 0.05 (Student’s *t* test). Bars depict the mean ± SEM of 6 wells. The graphs shown are representative of 2 experiments. (**E,F**) Representative gels are shown.

In order to evaluate whether PRLR was involved in cell migration as suggested by a previous study^12^, we evaluated the migration of GBM cells in a scratch assay. We observed that the blockade of PRLR using PRLR-A delayed wound healing in rat (C6, Fig. 5A) and human (LN229, Fig. 5B) GBM cells. Considering that the ability of tumour cells to migrate requires the activation of matrix metalloproteinases (MMP)^19^, we evaluated whether the activity of MMPs in GBM cell culture supernatant was stimulated by PRL. We found that MMP-2 was more abundant than MMP-9 in the cell supernatant of both rat and human GBM cells. PRL increased the content of activated MMP-2 in rat (C6; Fig. 5C,E; Supp. Fig. S2) and human (U251-MG; Fig. 5D,F; Supp. Fig. S3) GBM cell culture media. While MMP-9 was below detection threshold in treated and untreated rat GBM cell media, the addition of PRL increased the content of MMP-9 in human GBM cell conditioned media (Fig. 5D,F; Supp. Fig. S3).

### PRL and PRLR expression in GII-III and GBM patients

**Figure 6:**
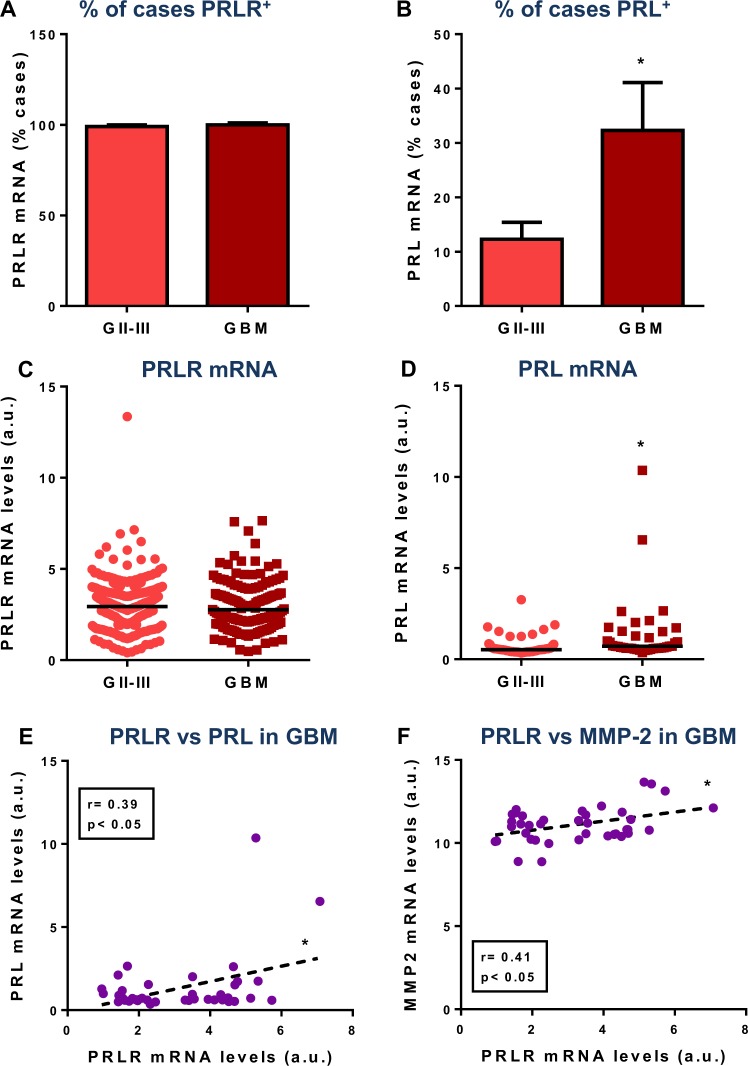
Transcriptomic analysis of prolactin and its receptor in human glioma. Meta-analysis of transcriptomic data from GII-III (n = 530) and GBM (n = 150) (The Cancer Genome Atlas): (**A,B**) % of samples expressing (**A**) PRLR mRNA or (**B**) PRL mRNA in GII-III (65/530) and GBM (45/150). * p < 0.05, χ^2^ test. (**C,D**) Dot plots showing the normalized expression of (**C**) PRLR mRNA levels and (**D**) PRL mRNA levels. *p < 0.05, Mann-Whitney U test. (**E,F**) Spearman correlation between (**E**) PRL and PRLR or between (**F**) MMP-2 and PRLR, in PRL^+^ GBM samples.

We next aimed to evaluate the expression of PRL and PRLR in human glioma samples. Meta-analysis of transcriptomic data from the TCGA revealed that PRLR is present in virtually all GII-III and GBM samples (Fig. 6A, Supp. Fig S4). PRL mRNA was detected in 12% of GII-III samples (65/530) and in significantly more GBM samples, with 30% of positive biopsies (45/150; Fig. 6B; Supp. Fig. S4). The levels of PRLR mRNA were similar in GII-III and GBM samples (Fig. 6C), while PRL mRNA levels were higher in GBM than in GII-III samples (Fig. 6D). Furthermore, in PRL-expressing GBM samples, PRLR mRNA expression levels had a significant correlation with PRL and also with MMP-2 mRNA expression levels (Fig. 6E,F).

**Figure 7:**
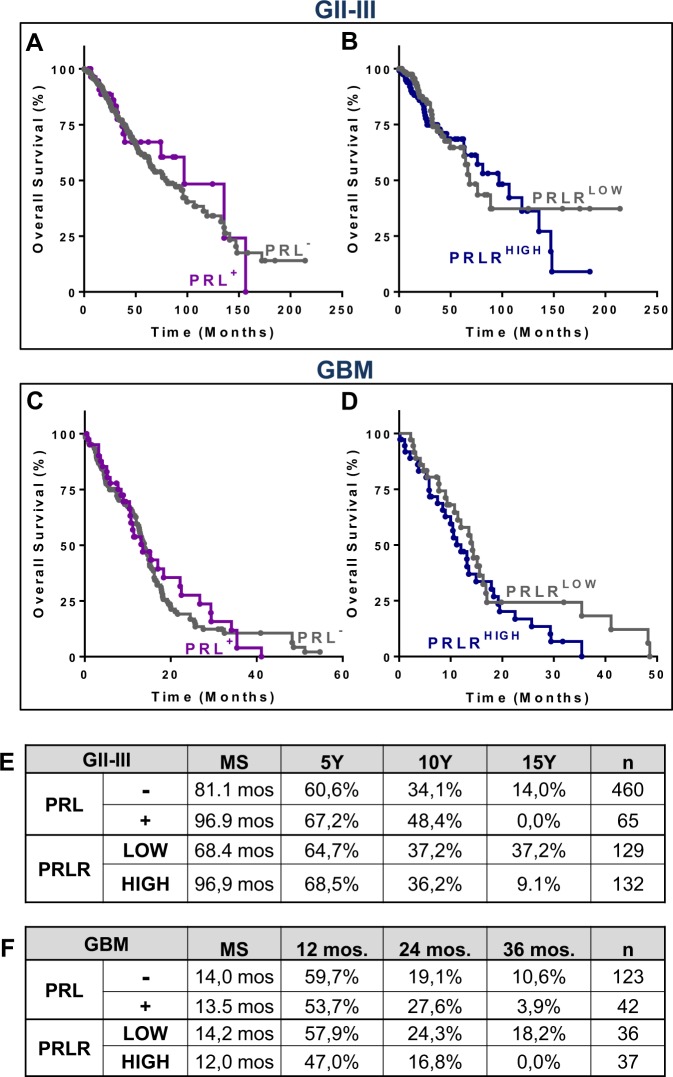
Survival of grade II-III glioma and glioblastoma multiforme patients according to the local expression of prolactin and its receptor. Meta-analysis of transcriptomic data from The Cancer Genome Atlas: (**A,B**) Kaplan-Meier survival curves of GII-III patients depending on the (**A**) tumour expression of PRL or (**B**) PRLR tumour expression levels (PRLR^HIGH^: 75% percentile; PRLR^LOW^: 25% percentile); (**C,D**) Kaplan-Meier survival curves of GBM patients depending on the (**C**) tumour expression of PRL or (**D**) PRLR tumour expression levels. (**E,F**) Tables show the median survival (MS), long-term survival and sample size of (**E**) GII-III and (**F**) GBM patients.

When we examined the survival of glioma patients according to the local expression of PRL or PRLR we found no significant changes, neither in the overall survival of PRL^+^ vs. PRL^−^ GII-III or GBM patients (Fig. 7A,C), nor in the overall survival of PRLR^HIGH^ vs. PRLR^LOW^ (Fig. 7B,D). However, the long-term survival rates of both GII-III and GBM patients that were PRL^+^ were lower than those with PRL^-^ tumours (Fig. 7E,F). In addition, glioma patients with PRLR^HIGH^ tumours exhibited lower long-term survival than those with PRLR^LOW^ tumours (Fig. 7E,F).

**Figure 8:**
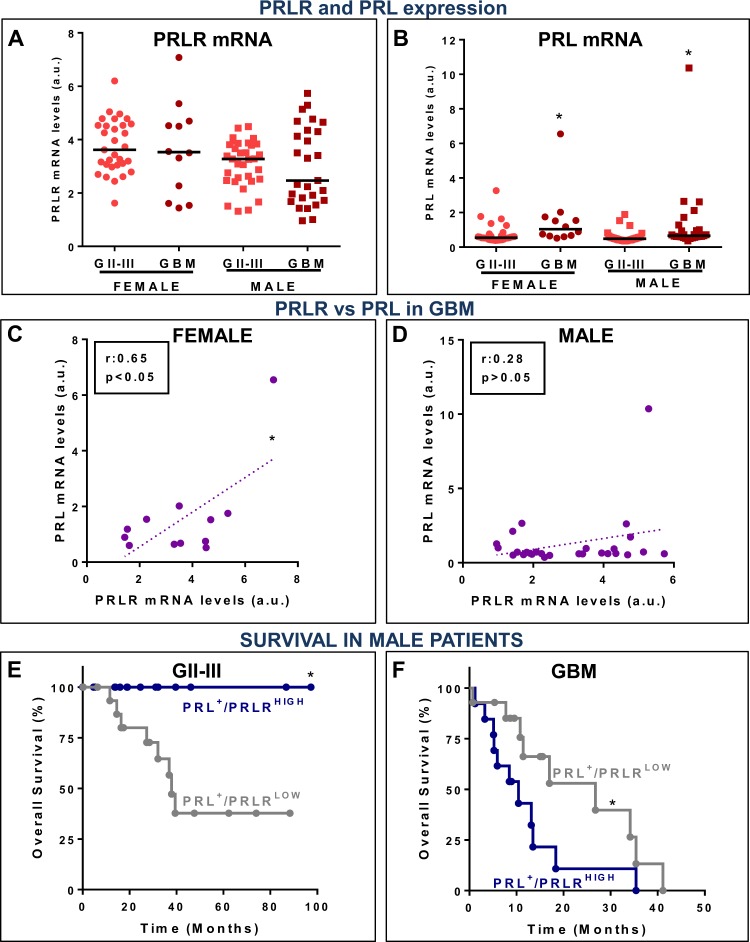
Transcriptomic analysis of prolactin and its receptor expression in glioma from female and male patients. Meta-analysis of transcriptomic data from female and male patients bearing GII-III or GBM (The Cancer Genome Atlas): (**A,B**) Dot plots showing the normalized expression of (**A**) PRLR mRNA levels and (**B**) PRL mRNA levels. *p < 0.05 vs. corresponding GII-III (Mann-Whitney U test), (**C,D**) Spearman correlation between PRL and PRLR mRNA in PRL^+^ GBM samples from (**C**) female and (**D**) male patients. (**E,F**) Kaplan-Meier survival curves of (**E**) GII-III and (**F**) GBM male patients that express PRL mRNA depending on PRLR mRNA expression levels. *p < 0.05 (Log-rank test).

The incidence of glioma has been reported to be significantly higher in male than in female patients^20^. This was also observed in the TCGA database that we analysed, where 55% GII-III patients (291/530) and 65% GBM patients (97/150) were males (Supp. Fig. S6A,B). No differences were observed in the median survival of female vs. male GII-III patients (F: 94.5 mos.; M: 81.1 mos.) or GBM patients (F: 14 mos.; M: 14 mos.) (Supp. Fig. S6C). The expression levels of PRLR mRNA did not show significant differences between GII-III and GBM patients in both cohorts of patients (Fig. 8A; Supp. Fig. S8A). The percentage of PRL^+^ samples in GII-III and GBM patients was similar between men [GII-III/PRL^+^: 12% (8–16%); GBM/PRL^+^: 28% (19–38%)] and women [GII-III/PRL^+^: 13% (9–18%); GBM/PRL^+^: 23% (12–36%)] and the upregulation of PRL mRNA levels in GBM with respect to GII-III samples was found in both female and male patients (Fig. 8B; Supp. Fig. S8B). However, the positive correlation between PRL and PRLR mRNA levels was only observed in female GBM patients (Fig. 8C,D; Supp. Fig. S9). We then analysed the survival of male PRL^+^ GII-III and GBM patients depending on their expression of PRLR (Fig. 8E,F). We found that male GBM patients that expressed high levels of PRLR exhibited worse survival (MS: 10.5 mos.) than those with low levels of PRLR (26.7 mos.) (Fig. 8F). Interestingly, we observed that male GII-III patients that expressed low levels of PRLR performed worse (MS: 37.98 mos.) than those with high PRLR, all of which were alive by month 100^th^ (Fig. 8E). While in female GII-III patients we did not observe differences depending on the expression of PRLR, there was a substantial decrease in the survival of female GBM patients with PRL^+^/PRLR^LOW^ vs. PRL^+^/PRLR^HIGH^, i.e. 6.6 mos. vs. 25.9 mos., respectively (p = 0.06, Log Rank test) (Supp. Fig. S7). This is an interesting observation as it is opposed to what we observed in male patients. Nevertheless, larger samples of female GBM specimens are required to draw conclusions on the correlation of PRLR with the survival of this subgroup of patients.

## Discussion

In this study we showed that PRL and PRLR were expressed by GBM cell lines and facilitated their viability, proliferation, clonogenicity, migration and chemoresistance. We detected PRL and PRLR protein expression in all GBM cell lines evaluated. PRL expression was also detected in human GBM xenografts, which was notorious in tumour cells infiltrating the non-neoplastic brain tissue. We observed homogenous expression of PRLR in U251-MG cells by immunofluorescence and WB, which revealed that human GBM cells express long, short and intermediate isoforms of PRLR, while rodent GBM cells seemed to express mainly the long isoform. PRLR was previously detected in U87-MG^12,21^ and U251-MG GBM cells^12^ and primary cultures of human GBM cells^21^. However, to best of our knowledge this is the first report depicting the isoforms of PRLR in GBM cells.

Invasion is a feature of GBM that plays a central on the pathogenesis of this tumour and seems to be involved in its high rate of recurrence. In agreement with previous reports from Alkharusi *et al*.^12^, our findings indicate that PRL facilitates GBM cell migration. Here we observed that PRL stimulation upregulates the activity of two matrix metalloproteinases (MMP-2 and MMP-9) in GBM cells, which are involved in tumour cell invasion and epithelial-to-mesenchymal transition (EMT)^22–25^. Wang *et al*.^23^ have shown a positive correlation between MMP-2 and MMP-9 expression and the malignancy of this tumour. A recent study showed that MMP-9 overexpression also promotes cell growth and increases the clonogenicity of human GBM cells^26^. Taken together, these findings suggest that PRL and PRLR would contribute to GBM cell migration, invasion and clonogenicity, partially by the modulation of MMP-2 and MMP-9 expression. In fact, analysis of transcriptomic data indicated that PRLR expression is positively correlated with the levels of MMP-2 expression in tumour samples from GBM patients.

Our results show that the activation of the PRL/PRLR pathway enhanced GBM cells chemoresistance to cisplatin and temozolomide. This process was already observed in hormone-dependent tumours, such as breast and prostate cancer^3,8,27–29^. PRL was found to abrogate cisplatin binding to DNA and to increase the sequestration of cisplatin in the cytoplasm by the glutathione-S-transferase^8^, whereas a PRLR antagonist sensitized T47D breast tumour cells to cisplatin^30^. Jak2/STAT5, the main signalling cascade activated by PRLR, and other cascades also triggered by PRLR, including MAPK (ERK1/2) and PI3K/Akt, have been linked to the pro-tumourigenic effects of PRL in hormone-dependent tumours, including cell proliferation, invasion, migration and chemoresistance^3^. In fact, STAT5 promotes the amplification of treatment-resistant prostate stem/progenitor cells, predicts early cancer recurrence and favours metastatic dissemination^3^. According to Alkharusi *et al*.^12^, PRLR activation also induces STAT5 phosphorylation in GBM cells, a mechanism that has been involved in tumour cell migration^12^. STAT5 pathway, which is the canonical pathway for long PRLR signalling^31^, was reported to be over-activated in GBM and directly involved in the modulation of proliferation, cellular transformation, migration and apoptosis^32–34^. Blockade of PRLR using different antagonists in GBM cells efficiently inhibited the phosphorylation of STAT5^12,35^. PRL was also reported to stimulate Ca^2+^ entry and its intracellular mobilization in GBM cells, together with a dose-dependent increase of GBM cell proliferation and viability^21^.

Studies evaluating the expression of PRL and PRLR in human GBM have generally analysed a small number of samples. However, the expression of these proteins has been consistently detected by immunofluorescence in 19%^36^, 25%^10^ and 47%^11^ of GBM biopsies. These figures are in agreement with the transcriptomic data that we analysed, which indicated that 30% of 150 GBM patients express PRL mRNA. PRLR was also detected by immunohistochemistry in GBM samples ranging from 44%^36^ to 66%^12^. Our analysis of transcriptomic data from GBM samples indicates that virtually all tumours express PRLR mRNA. In addition to the local detection of PRL and PRLR, hyperprolactinemia was also reported in 36%^11^ and 44%^36^ of GBM patients. Authors highlighted the fact that the proportion of hyperprolactinemia was twice as high in men as in women harbouring brain tumours, which opposes what happens in the general population, in which hyperprolactinemia is more frequent in women^36^, and reinforces the idea of sexual dimorphism in GBM pathogenesis. The development of hyperprolactinemia in GBM patients has also been proposed to be related to the irradiation of the hypothalamus during GBM treatment in a recent report that detected hyperprolactinemia in 32–35% of female and 66% of male patients^37^. Nevertheless, this growing body of evidence suggests that circulating levels of PRL should be closely monitored in glioma patients.

It has been proposed that the detection of PRL by immunohistochemistry in GBM specimens but not by real-time PCR indicates that the presence of PRL in primary tumours may not be a reflection of local production, but rather of circulating PRL that access the tumour^10^. However, the detection of PRL mRNA by GBM samples sequencing suggests that local production of PRL accounts, at least in part, for the presence of this hormone in GBM. Intracellular PRL has been shown to correlate with a high proliferation index in GBM biopsies, an effect that was associated with a mitogenic effect of this hormone^36^. Our findings using PRL and plasmids encoding PRL, which mimic the local expression of this hormone, suggest that PRL may exert a direct mitogenic effect in GBM cells. Moreover, male GBM patients that express local PRL and high levels of PRLR exhibited significantly worse overall survival than those with low local expression levels of PRLR. This finding supports the notion that local PRL/PRLR system plays a role in the pathogenesis of GBM facilitating tumour progression. In addition, while PRLR mRNA was present in virtually all GII-III and GBM biopsies at comparable levels, PRL mRNA expression was upregulated in GBM samples. This upregulation in PRL expression according to glioma grade was also observed when we stratified patient populations by their biological sex. Interestingly, the positive correlation between PRL and PRLR mRNA levels was only observed in female PRL^+^ GBM patients, suggesting that a positive feedback between these proteins may underlie sexual dimorphism. The sexual dimorphism in the incidence of GBM has been extensively reported, with a male/female ratio of ~1.6^20^, which is in agreement with the distribution of patients in the database we analysed (65% male vs. 35% female). Although we did not detect differences in the survival of male and female patients, a recent report indicates that the 5-year cancer-specific survival rate of female patients is significantly higher than in males^38^. This is in agreement with a recent report indicating that sexual dimorphism may also govern the response to treatment, as they observed that standard therapy is more effective in female GBM patients^39^.

Reproductive hormones have been proposed to influence the occurrence of GBM in the female population. A multi-centre study showed that the risk of developing glioma within the female population fluctuated with age at menarche, age at first parturition and hormonal contraceptive use^40^. While a case-control study found increased glioma risk in nulliparous women in comparison with parous women^41^, another study indicated that pregnancy was associated with tumour progression in female patients harbouring grade II-III, but not grade I tumours^42^. Long terms of breast-feeding were found to increase the risk of developing glioma when compared to shorter breast feeding periods^41^. Although circulating PRL may play a role in the pathogenesis of GBM, expression of PRL and PRLR in the tumour microenvironment may exert autocrine/paracrine effects that modulate GBM cell behaviour. Unfortunately, due to the relatively small number of female patients with PRL^+^ GBM, we could not perform a statistical analysis with enough power to conclusively compare the survival of female patients harbouring PRL^+^/PRLR^HIGH^ and PRL^+^/PRLR^LOW^ GBM. Nevertheless, the data shown here suggest that female patients with PRL^+^/PRLR^HIGH^ GBM live longer than those with PRL^+^/PRLR^LOW^ GBM. An opposite scenario was observed in the male PRL^+^ GBM population, in which the upregulation of PRLR was associated to a significant reduction in the median survival when compared to patients with PRLR^LOW^ GBM, who exhibited a striking 17-mos. difference in median survival.

Hormonal differences do not fully explain the differences between male and female GBM patients. It was previously reported that biological sex-specific differences in brain tumour rates are comparable at all ages, which implies that factors other than sex hormones are involved in these differences^43^. Sun *et al*.^43^ proposed that males are at higher risk of developing GBM due to an intrinsic sexual dimorphism in astrocyte transformation, as male GBM astrocytes exhibit higher proliferation rates, greater inactivation of the retinoblastoma tumour suppressor protein (RB), as well as increased tumourigenesis *in vivo* than female GBM astrocytes. Taken together, these results suggest that a proper treatment assignation according to biological sex differences may improve the care of GBM patients.

PRL was detected in a smaller percentage of GII-III patients and at lower expression levels than in GBM patients. Interestingly, we observed that male patients harbouring GII-III expressing PRL and high levels of PRLR had better survival than patients with low levels of PRLR. Strikingly, all male patients with PRL^+^/PRLR^HIGH^ GII-III were alive by month 100^th^. This result opposes our findings in GBM patients, but suggests that PRL and PRLR may hold value as therapeutic targets and/or prognostic biomarkers in both GII-III and GBM patients. Newly developed models of lower grade glioma^44^ may contribute to understand the role of PRL/PRLR in GII-III. Contradictory results on the role of PRL/PRLR have been also reported in hormone-dependent tumours. Although PRLR signalling has been traditionally involved in tumourigenesis of the mammary gland, it has been recently associated to the inhibition of breast cancer invasion, and a protective role of PRLR/STAT5 signalling has been proposed in already established tumours^3^. STAT5 activation seems to be necessary at the early stages of breast cancer and its phosphorylation seems to be lost during cancer progression (for a review see^3^). In fact, PRLR/STAT5 pathway has been shown to counteract EMT in human breast cancer cells hence to maintain them in a more differentiated, less aggressive state^45^. Summarizing, PRL/PRLR signalling may elicit very different outcomes depending on the biological sex of the patient and glioma grade, factors that need to be taken into consideration before translating therapies using PRLR antagonists to the neuro-oncology clinic.

In this study we detected PRL and PRLR expression across all GBM cell lines tested and showed that PRL/PRLR pathway is involved in their survival and response to chemotherapy. Even though GBM cell lines are commonly used by many researchers in translational neuro-oncology^46–51^, there are many publications that call into question the legitimacy of these *in vitro* models^52,53^. The utilization of serum in media may change the phenotype and/or genotype of GBM cell lines and cause depletion of stem cell-like tumour cells^53,54^. Furthermore, the injection of these cells for *in vivo* GBM models may fail to accurately mirror important morphological features of the tumour^53–57^. Additionally, differences between serum batches can disrupt reproducibility^58^. Therefore, glioma neurospheres^59–61^, annotated and validated cell lines derived from surgical samples of GBM patients^53,55,59,62^ and other serum-free cell cultures^54^ should be considered for future prospects in order to perform experiments that reflect more realistically the gliomas’ microenvironment. Moreover, further development of lower grade glioma models is required to clarify why the activation of PRL/PRLR signalling elicit opposite outcomes between GII-III and GBM patients. Our study proposes PRLR as a therapeutic target for the treatment of GBM and warrants further evaluation of PRL and PRLR as prognostic biomarkers in glioma patients. Our work provides additional evidence to the notion that sexual dimorphism should be taken into account to improve the care of GBM patients.

## Methods

### Patients and datasets

RNA-Seq expression data of PRL, PRLR and MMP-2 (Illumina HiSeq 2000 RNA sequencing platform) from 530 GII-III samples and 150 GBM samples were obtained from The Cancer Genome Atlas (TCGA) and analysed using UCSC Xena browser (10.1101/326470)^63^. This database provides quantitative gene expression information and a compelling list of patients’ characteristics, including their clinical parameters and survival rates (Supp. Fig. S5).

### Drugs

Dulbecco’s Modified Eagle’s Medium (DMEM; 12800017), Penicillin-Streptomycin, Trypsin-EDTA (0.05%) and Lipofectamine 2000 were obtained from Gibco (Invitrogen, Carlsbad, CA); fetal bovine serum (FBS) and horse serum (HS) from Natocor (Córdoba, Argentina); cisplatin from Microsules (Buenos Aires, Argentina); and temozolomide and ovine prolactin from Sigma (St. Louis, MO). OCT medium for frozen sections was obtained from Biopack (Buenos Aires, Argentina). Ketamine was obtained from Holliday (Argentina Poniente, Mexico). Xylazine (Kensol) was obtained from König (Buenos Aires, Argentina). Ketoprofen (Ketofen) was from Merial Laboratories S.A. (Buenos Aires, Argentina). Anti-rat PRL is from Dr. A. Parlow, National Hormone and Pituitary Program (NHPP; Torrance, CA). The PRLR antagonist ∆1–9-G129R-hPRL was produced by recombinant technology and purified by ion exchange chromatography as previously described^17^. Anti-human PRL (A0569) was obtained from Dako (Santa Clara, CA) and anti-human PRLR antibodies for WB and immunocytochemistry (H-300 and D-7) are from Santa Cruz Biotechnology (Dallas, TX). Anti-rabbit IgG and anti-rabbit fluorescein-conjugated secondary antibody are from Vector Laboratories Inc. (Burlingame, CA). Anti-guinea pig rhodamine-conjugated secondary antibody is from Chemicon International (Temecula, CA).

### Cell culture

GBM cell lines were grown in Petri dishes containing DMEM with high glucose, L-glutamine, sodium pyruvate and sodium bicarbonate, supplemented with 10% SFB and 1% Penicillin-Streptomycin. Cells were harvested using Trypsin-EDTA (0.05%) in PBS and counted with Trypan-blue. For *in vitro* experiments, cells were grown with 5% HS in order to avoid any possible interaction between bovine PRL from FBS and cells’ PRLR. For the experiments, the doses of PRL (100 ng/ml)^8,12,21,64–66^ and PRLR-A (2.5 µg/ml)^7,17,18,67–70^ were obtained from previous publications. Dose selection for cisplatin and TMZ can be found in Supp. Fig. S10.

### Animals

Adult female athymic N:NIH Swiss mice (6–8 week old) were purchased at the vivarium of Facultad de Ciencias Veterinarias, Universidad Nacional de La Plata, Argentina, and kept in controlled conditions of light (12 h light-dark cycles) and temperature (20–25 °C). Mice were fed with standard lab chow and water *ad libitum* and all efforts were made to minimize distress. All animal work was conducted according to the NIH guidelines and was approved by the Institutional Ethical Committee (*Comité Institucional para el Cuidado y Uso de Animales de Laboratorio*, CICUAL), Facultad de Medicina, Universidad de Buenos Aires; approval ID: Res. (CD) N° 697/19.

### Brain tumour models

Nude intracranial tumour models were generated as previously described^71^. Briefly, mice were anesthetized with ketamine (100 mg/kg) and xylazine (15 mg/kg) and placed in a stereotactic apparatus modified for mice. Human U251-MG GBM cells (1.5 × 10^6^ cells) were injected in a volume of 5 µl unilaterally into the right striatum (+0.5 mm AP; −2.1 mm ML; −2.9, −3.2, −3.5, −3.8, −4.1 mm DV from bregma) using a 5 µl Hamilton syringe with a 33-gauge needle. Mice received ketoprofen analgesic (4 mg/kg) the day of the surgery and the next day. 35 days after injection, mice were perfused using Tyrode’s buffer and 4% paraformaldehyde (PFA) and brains were collected immediately and processed for immunohistochemistry.

### Immunocytochemistry

After perfusion, brains were post-fixed for 72 h in 4% PFA, washed with PBS, soaked in cold 20% sucrose overnight, frozen to −70 °C with 2-methylbutane (isopentane) in a dry ice/acetone bath, and finally sectioned in cryostat using OCT freezing media. Tumour cells seeded in coverslips were fixed with 4% PFA for 10 min on ice and washed with PBS. For immunocytochemistry against rat and human PRL, cells were permeabilized in citrate buffer (pH 6) at 350 W for 7 min., followed by washing during 5 min. with TBS- 0.5% Triton- 0.1% Azide. Blockade was performed in TBS- 0.2% Triton- 0.1% Azide- 10% goat serum for 1 h and incubation with antibodies against rat PRL (NHPP) or human PRL (A0569, Dako) was performed overnight in TBS- 0.2% Triton- 0.1% Azide- 1% goat serum. Immunocytochemistry for human PRLR was performed without citrate buffer permeabilization. Brain sections and cells were blocked with PBS- 10% goat serum for 1 h and incubation with antibody against human PRLR (sc-20992, Santa Cruz Biotechnology) was performed overnight in PBS− 1% goat serum. Then, cells and tissues were incubated with their respective fluorescent secondary antibodies, anti-guinea pig (Chemicon International) or anti-rabbit (Vector Laboratories), for 1.5 h. After washing with distilled water, cells and tissues were incubated with DAPI for 10 min, washed and mounted on slides using Vectashield (Vector Laboratories). Negative controls were incubated without the primary antibodies. For the immunocytochemistry of PRLR, somatolactotrope GH3 cells were used as the positive control. The specificity of the antibodies used here was previously reported for PRLR^72^ and PRL^73^.

### Western Blot (WB)

Total proteins were extracted from GBM cell lines’ cultures with NP-40 lysis buffer containing 150 mM NaCl, 50 mM Tris Base (pH 8), 1% Triton X-100 in water, and a protease inhibitor cocktail (1/100; P8340, Sigma). Following centrifugation at 12,000 g for 40 min, the supernatant was recovered. Protein concentration of each sample was determined by Bradford protein assay (Bio-Rad Laboratories, Hercules, CA). 40 µg of protein were size-fractionated in 12% SDS-polyacrylamide gel and then electrotransferred to polyvinyl difluoride (PVDF) membranes. Correct protein loading and transfer efficiency were assessed by membrane staining with red Ponceau. Blots were blocked for 120 min in 5% non-fat dry milk- PBS- 0.1% Tween 20 at room temperature and incubated overnight at 4 °C with anti-PRLR antibody (H300, 1/250, and D7, 1/200; Santa Cruz) in the same buffer. After washing, membranes were incubated for 1 h with HRP-conjugated anti-rabbit (1/1000; Millipore, Cat# AP103P) or anti-mouse antibody (1/1000; Millipore, Cat# AP130P) in 5% non-fat dry milk- PBS- 0.1% Tween 20. Blots incubated in the absence of primary antibody were used as negative controls. Immunoreactivity was detected by enhanced chemiluminescence (Productos Bio-Lógicos, Argentina) in a chemoluminiscence imaging system (G Box Chemi HR16, Syngene; Cambridge, UK).

### PRL radioimmunoassay

PRL was measured in C6 and U251-MG cell extracts and supernatants. Cells were seeded in 24-well plates and cultured in 200 μl/well for 48 h. Then, supernatants were collected and cell proteins were extracted as described above. PRL levels were measured by RIA using reagents provided by the National Institute of Diabetes and Digestive and Kidney Diseases, National Hormone and Pituitary Program (Torrance, CA) as previously described^69^.

### BrdU incorporation assay

Human GBM cells were incubated with 100 ng/ml ovine PRL or 2.5 µg/ml PRLR-A for 6 h. Then, cells were incubated with 10 µM BrdU labelling solution for the last 1.5 h and BrdU incorporation into cellular DNA strands was assessed by ELISA following manufacturer’s instructions (Roche Molecular Biochemicals, Mannheim, Germany; Cat# 11647229001) as previously described^74^. Absorbance was measured in 96-well plate spectrophotometer (Bio-Rad, Model 550) at 450 nm.

### Transfections

Human U251-MG cells were seeded in 24-well plates and 24 h later were transfected for 6 h with plasmids encoding human prolactin (pPRL) under the control of the cytomegalovirus (CMV) promoter or encoding the PRLR-A (pPRLR-A) under the control of the metallothionein promoter^75^, using Lipofectamine 2000 (Invitrogen) following the indications of the supplier. Controls were incubated with an empty pcDNA3 plasmid (pCTRL). After 16 h, 5 µM cisplatin was added to the incubation media for additional 72 h, and cell viability was finally assessed by MTT assay.

Mouse GBM GL26 cells were transfected as described above with plasmids encoding the short (pSPRLR) or the long (pLPRLR) isoforms of the mouse PRLR under the control of the EF1 promoter that were kindly donated by Dr Julia Halperin (Universidad Maimonides, Buenos Aires, Argentina)^76,77^. 2 µM cisplatin was added to the incubation media for additional 72 h, and cell viability was finally assessed by MTT assay. Rat GBM C6 cells were transfected with plasmids encoding the short (pSPRLR) or the long (pLPRLR) isoforms of the rat PRLR under the control of the CMV promoter^78^. After 16 h, cells were incubated with 1.5 µM cisplatin and processed for MTT or clonogenic assays.

### Cell viability assay (MTT)

Cell viability was analysed using 3-(4,5-dimethylthiazol-2-yl)−2,5-diphenyltetrazolium bromide (MTT; Molecular Probes, Invitrogen) as described before^74^. Absorbance was determined using a 96-well plate spectrophotometer (Bio-Rad, Model 550) at 595 nm.

### Clonogenic assay

After transfecting rat GBM C6 cells with pCTRL, pSPRLR or pLPRLR, cisplatin (1.5 µM) was added to the medium for 24 h. Then, cells were harvested with trypsin and 450 cells were seeded in 6-well tissue culture plates. Ten days later, cells were stained with Giemsa. The number of colonies containing a minimum of 50 cells (colony-forming unit, CFU) was counted under microscope.

### Scratch assay

Rat GBM C6 cells and human GBM LN229 cells were seeded with or without PRLR-A in 24-well plates for 24 h. A wound was then performed by scratching the confluent cell culture wells with a micropipette tip. Afterwards, cells were washed with PBS and reincubated with or without PRLR-A in complete DMEM without serum. Lastly, cells were photographed at different time-points for up to 10 h and the wound area was measured using ImageJ Software.

### Zymography

Rat C6 and human U251-MG GBM cells were incubated for 48 h in the presence of 100 ng/ml PRL. Conditioned media was collected and MMP gelatinocytic activity was assessed by zymography. 3 μl of medium was loaded onto 10% acrylamide gel containing 0.2% gelatine and run at 120 V. Gels were then washed with 50 mM Tris-HCl pH 7.5 in 2.5% Triton X-100 for 45 min, followed by a 45 min washing step with a 50 mM Tris HCl solution containing 5 mM CaCl_2_ and 1 µM ZnCl_2_ plus 2.5% Triton X-100, pH 7.5. Gels were then incubated for 24 h at 37 °C with a solution of 50 mM TrisHCl containing 10 mM CaCl_2_, 200 mM NaCl, pH7.5. Finally, gels were stained with 0.5% Coomassie Brilliant Blue R-250 and destained with decolorizing solution (25% v/v isopropanol plus 10% v/v acetic acid). Enzyme activity, seen as clear bands against a blue background was analysed by densitometry with ImageJ image processing system. The zymographic activity was expressed as a percentage in relation to a standard internal sample that saturates at a density of 50%. Data corresponding to different gels were normalized using internal control samples.

### Statistical analysis

Data were graphed and analysed using GraphPad Prism version 5 software (GraphPad Software). All the data were tested for normality using the Kolmogorov Smirnoff test before performing parametric statistical tests. The data obtained using cell lines were normally distributed. Differences in BrdU incorporation, clonogenic ratio and MTT data were analysed by analysis of variance (ANOVA) followed by Tukey’s post-test. Differences in the expression levels of PRLR and PRL mRNA were assessed by Student’s *t* test, whereas the proportion of positive vs. negative patients was analysed by χ^2^ test. Nonlinear correlation analysis was used to analyse differences in the scratch assay. Correlation between PRL and PRLR expression was evaluated by Pearson analysis. Kaplan-Meier survival values were calculated for the low and high expression of PRL and PRLR. Overall survival between the groups was compared using log-rank test. Differences between groups were considered significant when p < 0.05. All the experiments were performed at least twice.

### Statement of ethics

All animal procedures were conducted according to the NIH guidelines and approved by the Institutional Ethical Committee (Comité Institucional para el Cuidado y Uso de Animales de Laboratorio, CICUAL) of the Facultad de Medicina, Universidad de Buenos Aires; approval ID: Res. (CD) N° 697/19.

## Supplementary information

Supplementary Figures
