# Supplementary material for: Prolactin and its receptor as therapeutic targets in glioblastoma multiforme
Source: Sci Rep. 2019 Dec 20;9:19578. doi: 10.1038/s41598-019-55860-x (PMC6925187; doi:10.1038/s41598-019-55860-x)
Supplement: Supplementary file 1 — Supplementary Figures [file 41598_2019_55860_MOESM1_ESM.pdf]

Prolactin and its receptor as therapeutic targets in glioblastoma multiforme

Antonela Sofía Asad, Alejandro Javier Nicola Candia, Nazareno Gonzalez, Camila Florencia Zuccato, Araceli Abt, Santiago Jordi Orrillo, Yael Lastra, Emilio De Simone, Florence Boutillon, Vincent Goffin, Adriana Seilicovich, Daniel Alberto Pisera, María Jimena Ferraris, Marianela Candolfi*

*Corresponding Author: mcandolfi@fmed.uba.ar

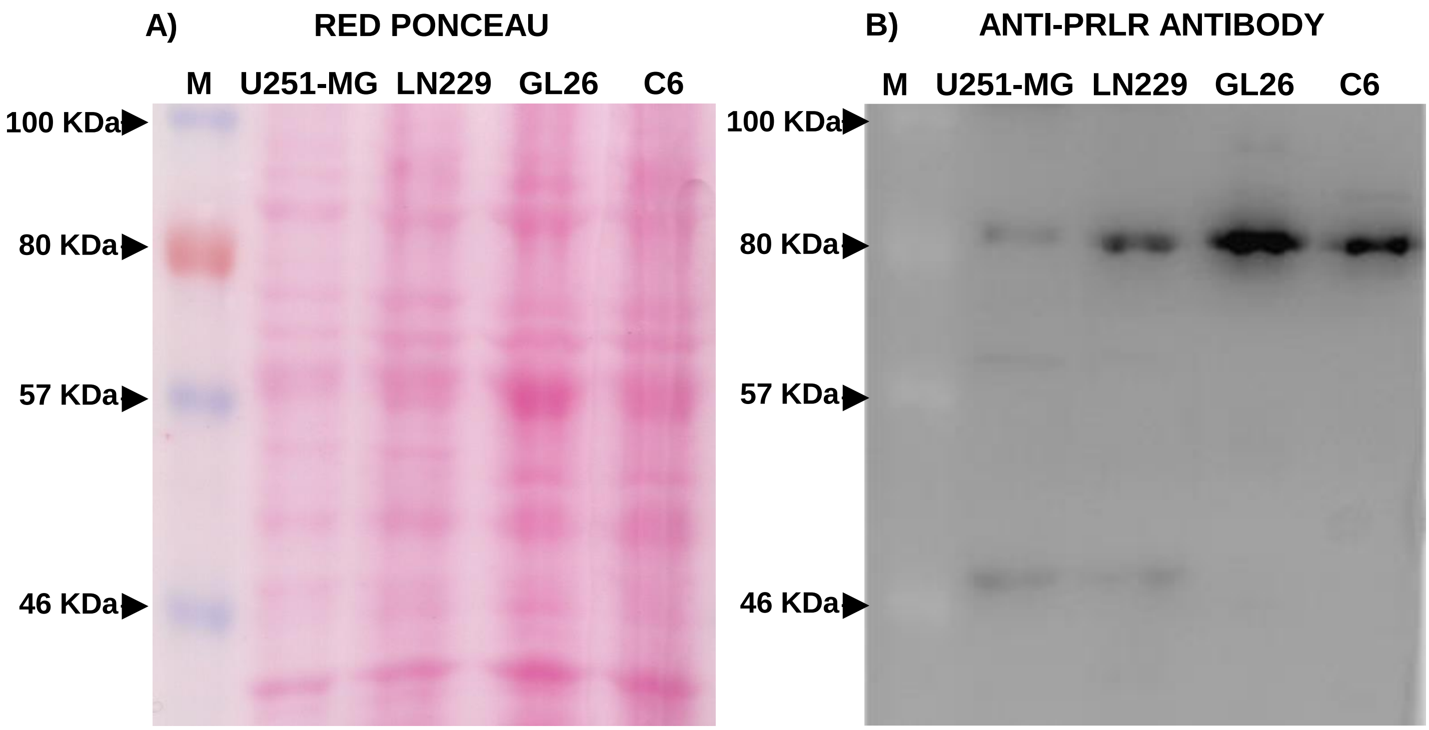

Supplementary Figure S1: Full-length gels show the expression of PRLR in human and rodent GBM cell lines. A representative Western blot gel of protein extracts from human (U251-MG, LN229), murine (GL26) and rat (C6) GBM cell lines stained with (**A**) Red Ponceau or (**B**) anti-PRLR antibody. M: molecular weight marker.

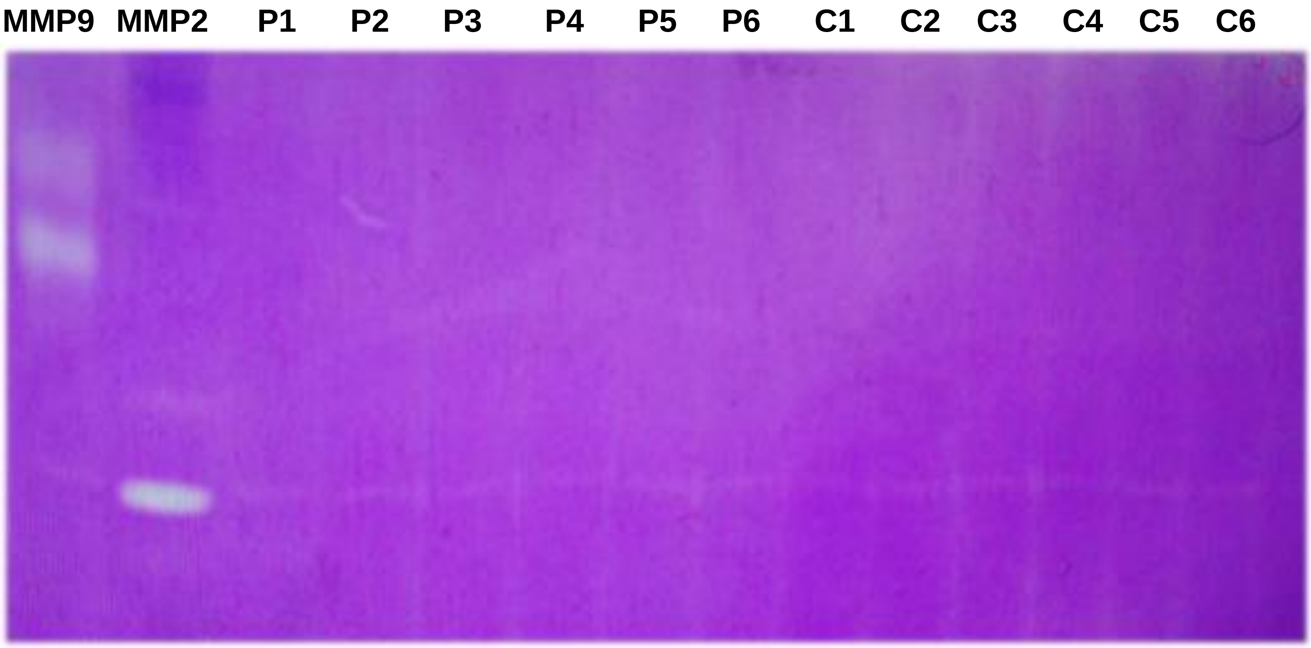

Supplementary Figure S2: Full-length gel shows MMP-9 and MMP-2 activity in rat GBM cells. SDS-PAGE gelatine zymography of conditioned media from rat C6 GBM cells incubated in the absence (C1-C6) or presence (P1-P6) of PRL (100 ng/ml) for 48 h. Gels were stained with Coomassie blue and bands were analysed by densitometry with ImageJ software. Zymographic activity was expressed as percentage in relation to a standard internal sample (MMP-9 and MMP-2) that saturates at a density of 50%.

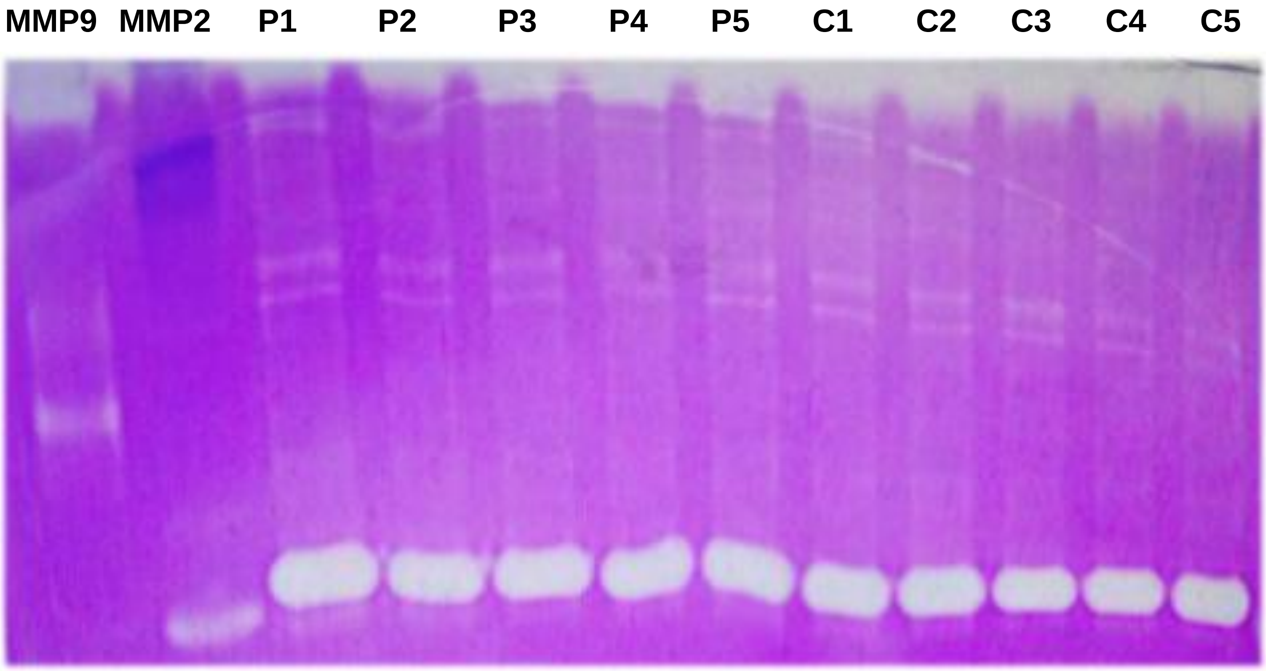

Supplementary Figure S3: Full-length gel shows MMP-9 and MMP-2 activity in human GBM cells. SDS-PAGE gelatine zymography of conditioned media from human U251-MG GBM cells incubated in the absence (C1-C5) or presence (P1-P5) of PRL (100 ng/ml) for 48 h. Gels were stained with Coomassie blue and bands were analysed by densitometry with ImageJ software. Zymographic activity was expressed as percentage in relation to a standard internal sample (MMP-9 and MMP-2) that saturates at a density of 50%.

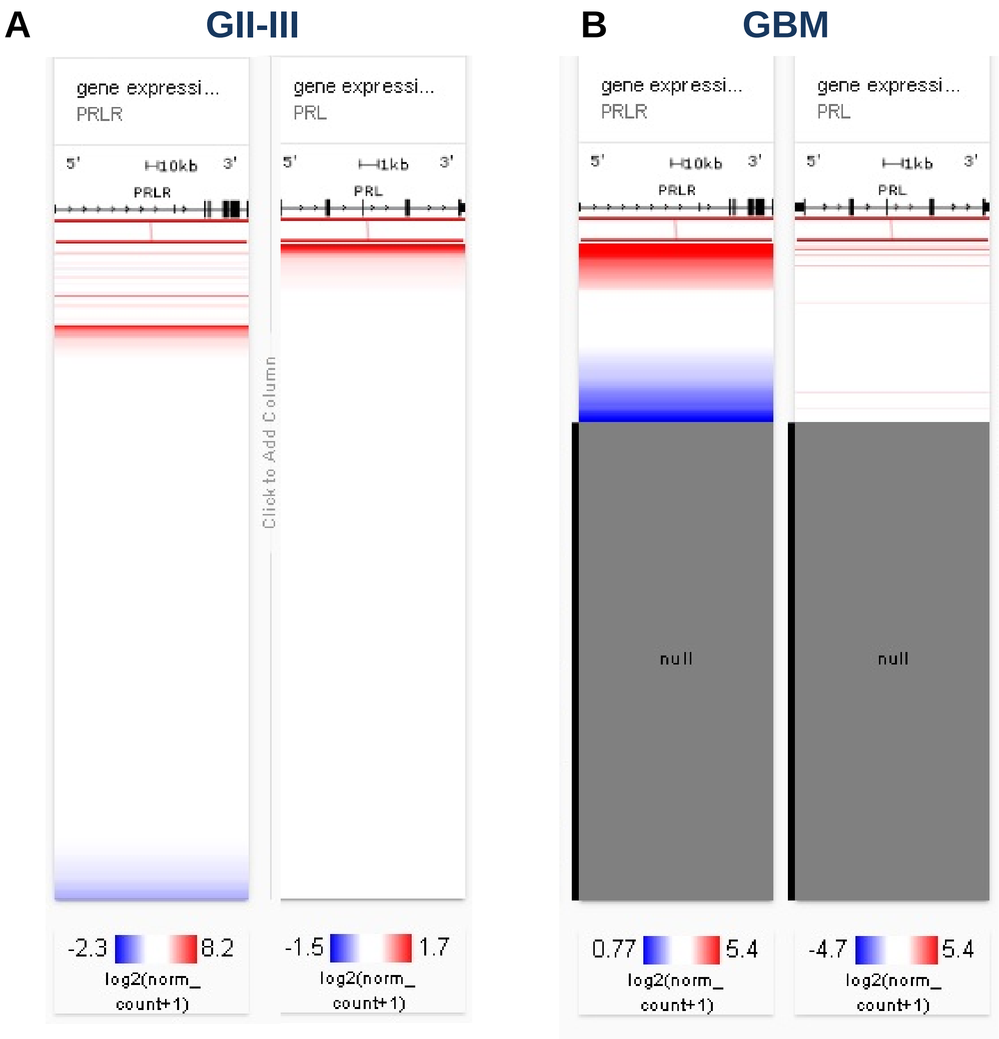

Supplementary Figure S4. PRLR and PRL gene expression heatmaps of GII-III and GBM samples from TCGA. PRLR and PRL gene expression data were available for (**A**) all GII-III samples (n=530) and (**B**) a fraction of GBM samples (n=130). In grey, GBM samples in which PRLR and PRL gene expression data were not available (n=501).

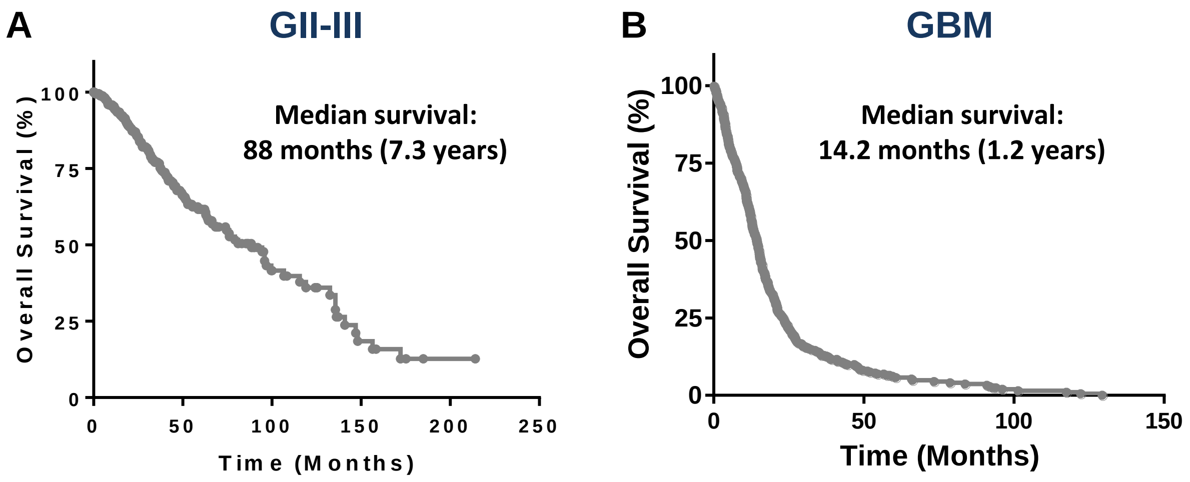

Supplementary Figure S5. Survival of glioma patients from TCGA. Kaplan-Meier survival curves of (**A**) GII-III and (**B**) GBM patients, as assessed by meta-analysis of transcriptomic data from The Cancer Genome Atlas (TCGA).

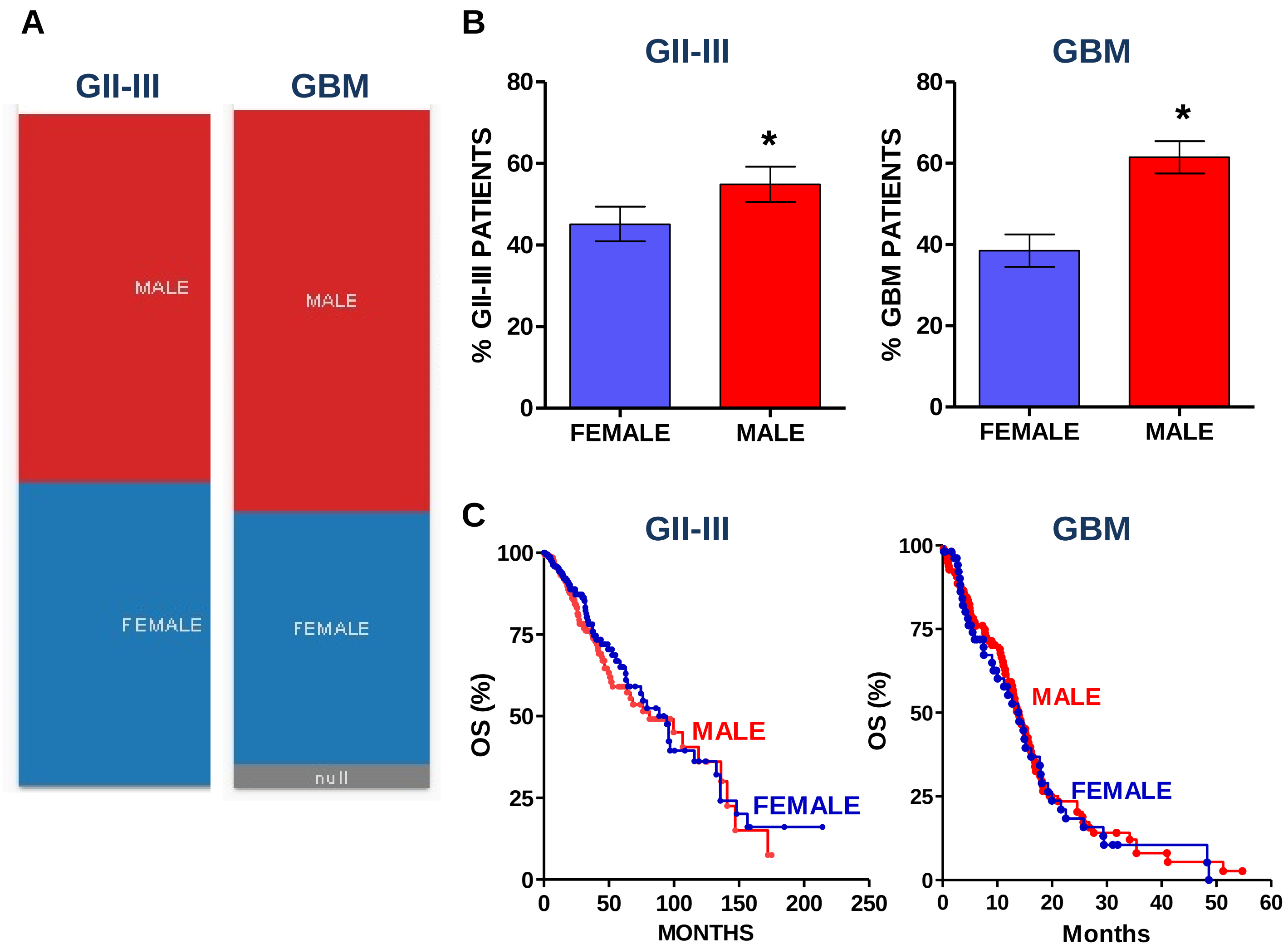

Supplementary Figure S6. Distribution of GII-III and GBM samples from TCGA according to patient's biological sex. A) Graphs depict the distribution of female and male patients harbouring GII-III (n=530) and GBM (n=631). In grey, GBM samples in which the information on biological sex was not available (n=21). **B**) Graphs showing the mean±confidence intervals of glioma male and female proportions. *p<0.05 (Chi^2 test). **C**) Overall survival (OS) of female vs. male GII-III and GBM patients.

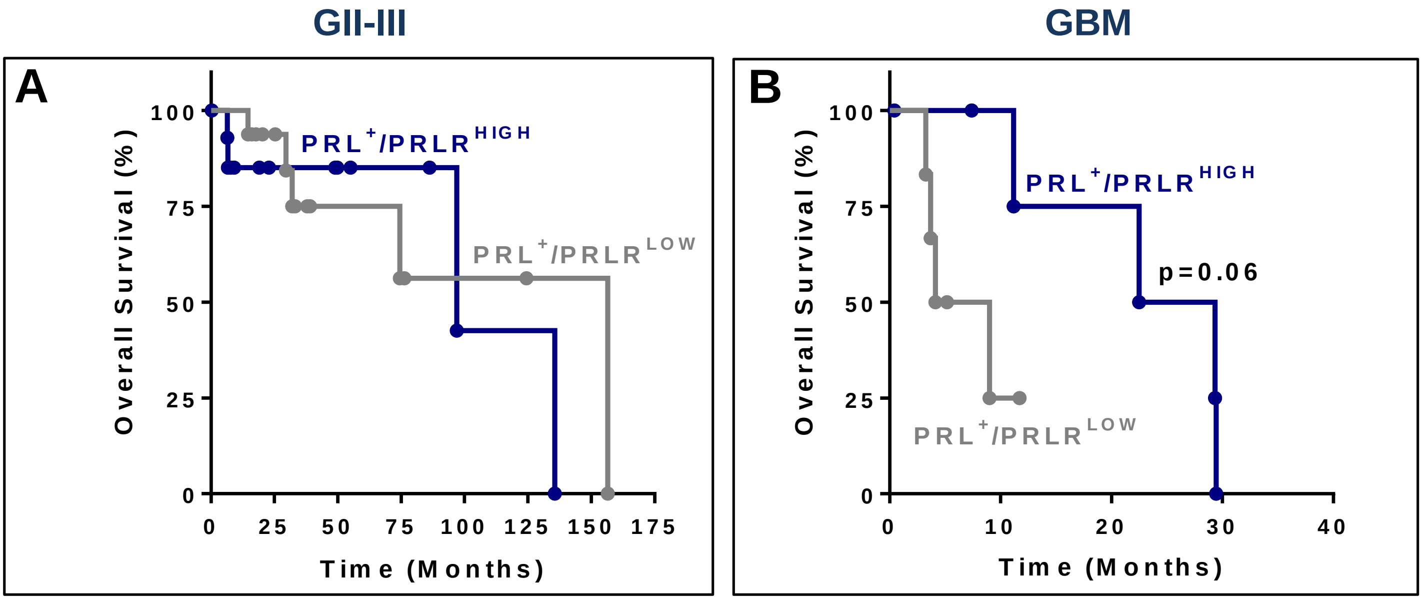

Supplementary Figure S7. Survival of female PRL$^+$ glioma patients according to the expression of PRLR. Kaplan-Meier survival curves of (**A**) GII-III (n=15-16/group) and (**B**) GBM (n=6/group) female patients that express PRL mRNA depending on PRLR mRNA expression levels. p=0.06, Log-rank test.

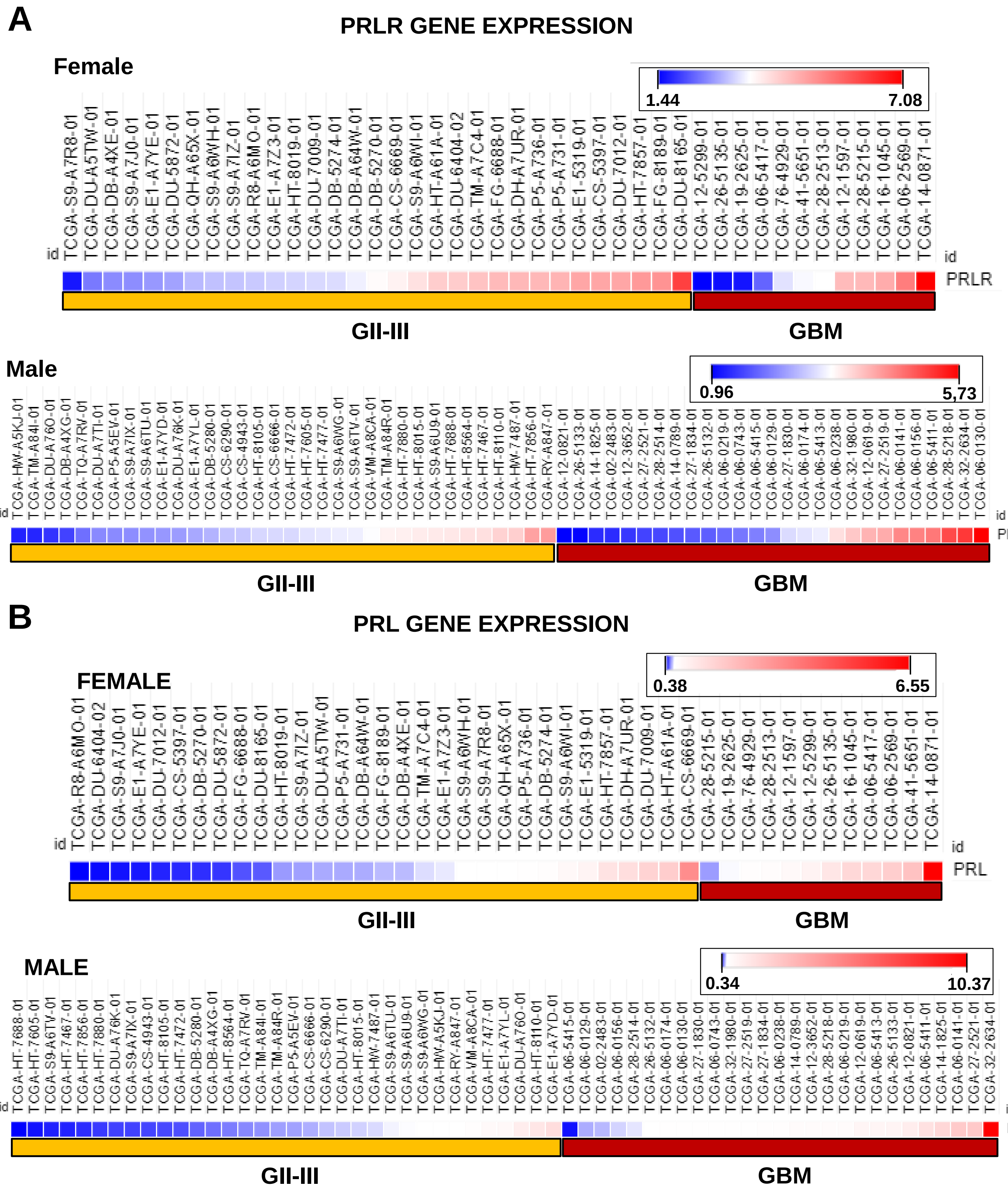

Supplementary Figure S8. Transcriptomic analysis of PRLR and PRL gene expression in GII-III or GBM from female and male patients (TCGA). Heatmaps show the normalized expression of (**A**) PRLR and (**B**) PRL genes in GI-III and GBM from female and male patients.

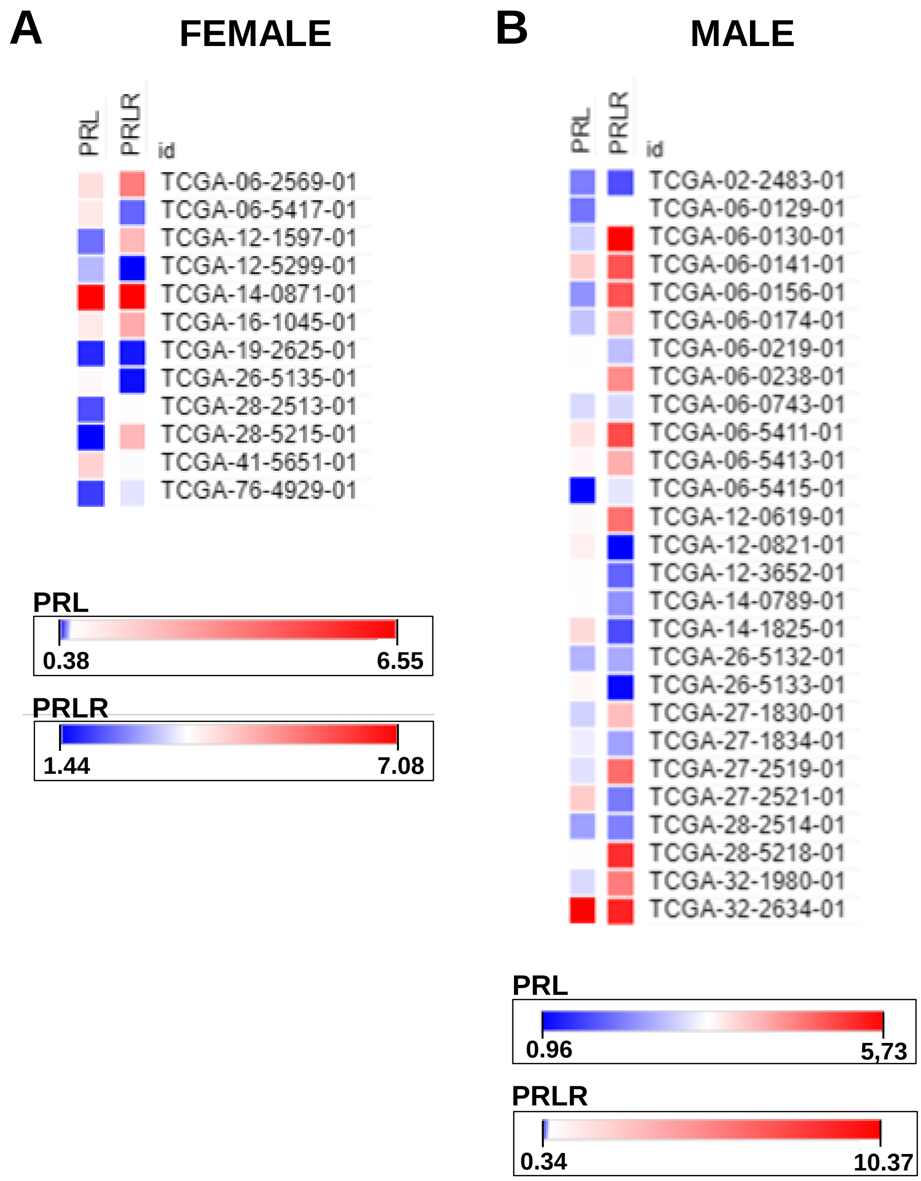

Supplementary Figure S9. Meta-analysis of PRL and PRLR gene expression in GBM from female and male patients (TCGA). Heatmaps of PRL and PRLR mRNA in PRL-expressing GBM samples from (**A**) female and (**B**) male patients.

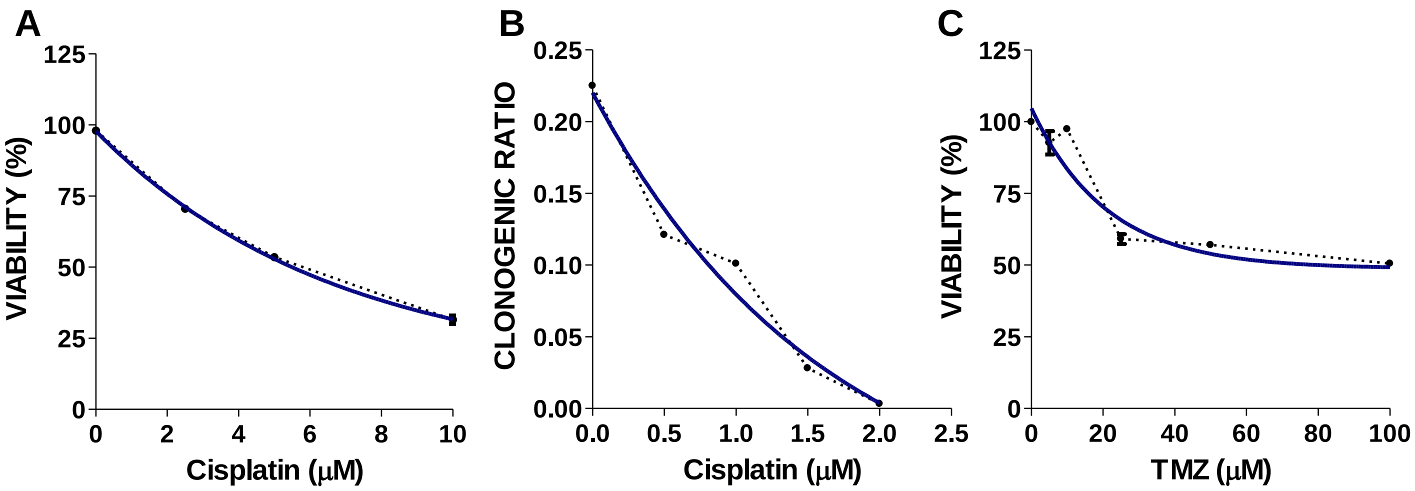

Supplementary Figure S10. Chemotherapeutic drug dose-response curves in GBM cells. A) U251-MG GBM cells were incubated with different doses of cisplatin for 72 h. Cells were then processed for the clonogenic assay. **B,C**) U251-MG cells were incubated with increasing doses of (**B**) cisplatin or (**C**) TMZ for 72 h. Cell viability was assessed by MTT assay. Dotted lines connect the mean ± SEM at each concentration. Full lines: multiple regression analysis (R^2: **A**: 0,97; **B**: 0,99; **C**: 0,91).
